# Impact of standard precautions and unrestricted movements of carbapenemase-producing *Enterobacterales* (CPE) carriers on CPE transmission in a nursing home in Singapore: a prospective cohort study

**DOI:** 10.1186/s13756-025-01554-1

**Published:** 2025-04-28

**Authors:** Kyaw Zaw Linn, Xiaowei Huan, Pei Yun Hon, Sharifah Farhanah Syed Husen, Natascha May Thevasagayam, Oon Tek Ng, Shawn Vasoo, Moi Lin Ling, Dale Fisher, Kalisvar Marimuthu

**Affiliations:** 1 Communicable Diseases Agency, Singapore, Singapore; 2 National Centre for Infectious Diseases, Singapore, Singapore; 3 Tan Tock Seng Hospital, Singapore, Singapore; 4 Lee Kong Chian School of Medicine, Nanyang Technological University, Singapore, Singapore; 5 Department of Infectious Diseases, The National Centre for Infectious Diseases and Tan Tock Seng Hospital, Singapore, Singapore; 6 Yong Loo Lin School of Medicine, The National University of Singapore, Singapore, Singapore; 7 Infection Prevention and Epidemiology, Singapore General Hospital, Singapore, Singapore; 8 Department of Medicine, National University Hospital, Singapore, Singapore, Singapore

**Keywords:** Carbapenem-resistant *Enterobacterales*, CRE, Carbapenemase-producing *Enterobacterales*, CPE, Standard precaution, Long-term care facility

## Abstract

**Background:**

In 2018, Singapore’s National Infection Prevention & Control Committee (NIPC) recommended standard precautions and unrestricted movements for CPE carriers in nursing homes.

**Objective:**

This study investigates the short-term impact of this intervention on CPE transmission in a nursing home in Singapore.

**Methods:**

We conducted a prospective cohort study between 1st April and 11th July 2019 in a 255-bedded nursing home in Singapore. Stool samples from residents and environmental samples from sink strainers in the residents’ bedrooms, bathrooms, and lavatories, and shower drain traps in bathrooms were collected at baseline, week 2, week 8, and week 12 and tested for CPE. We performed whole genomic sequencing (WGS) to find out if there was any bacterial or plasmid linkage among the residents and between the residents and environment.

**Results:**

A total of 32 residents, including six known CPE carriers, were recruited and completed the three-month follow-up visits. Of the six known CPE carriers, five tested negative for CPE, while one consistently tested positive for CPE throughout the study. Of the 28 sink strainers, six (21.43%) were positive for CPE. CPE was not detected in any shower drain trap throughout the study. Only one resident acquired CPE at week 12. WGS analysis of available CPE isolates showed no bacterial or plasmid linkage between residents or between residents and the environment.

**Conclusions:**

Standard precautions and unrestricted movement of CPE carriers may be sufficient to control CPE transmission in the nursing home setting. Larger studies with more extensive environmental sampling and longer follow-up periods are needed to confirm this.

**Supplementary Information:**

The online version contains supplementary material available at 10.1186/s13756-025-01554-1.

## Introduction

Outbreaks of carbapenemase-producing *Enterobacterales* (CPE) in nursing homes highlight the need for infection prevention and control (IPC) measures [1, 2]. People are often identified as colonized by CPE during surveillance programmes in Singapore’s acute hospitals. Implementing contact precautions with isolation for CPE carriers in nursing homes poses practical challenges and may negatively impact residents’ well-being. IPC guidelines for nursing homes differ in their recommendations for CPE carriers [3–6]. In 2018, Singapore’s National IPC Committee recommended standard precautions and unrestricted movements for CPE carriers in nursing homes [7]. This pilot study investigates the short-term impact of this intervention on CPE transmission in a nursing home in Singapore.

## Method

### Study design and setting

We conducted a prospective cohort study between 1st April and 11th July 2019 in a 255-bedded nursing home in Singapore. There were 3 floors where nursing home residents were housed. On each floor, there were eight 9-bedded rooms and two 7-bedded rooms. We excluded one floor that housed only dementia patients, as approaching the next of kin of the residents on the entire floor would be logistically infeasible. The floor that housed female residents had one room where known CPE carriers were housed as a cohort and one room where methicillin resistant Staphylococcus aureus (MRSA) carriers were housed as cohort. The floor that housed male residents did not have any cohort rooms. The layout of the floors, including the bathrooms and sinks, can be seen in Figure S1 and S2. Each cubicle has one sink in the residents’ bedroom, one sink and one shower drain trap in the bathroom, and one sink in the lavatory. The lavatories on the floor that housed female residents were used as storerooms by staff and were not used by residents. Five out of the seven known CPE carriers were placed in a CPE cohort cubicle, and the remaining two were placed in another cubicle. The movement of the CPE carriers and their contact with other residents were not restricted.

We excluded residents who were deemed unsuitable for the study by the nursing team, as well as those who were unable to provide consent or for whom approaching their next of kin or legal guardian would be logistically infeasible. Stool samples from residents, and environmental samples from sink strainers in the residents’ bedrooms, bathrooms, and lavatories, and shower drain traps in the bathrooms were collected at baseline, week 2, week 8, and week 12 (Supplementary Table S1) using Copan Elution Swabs (eSwab^®^). The swabs were pre-moistened with sterile water and gently rubbed across the surface of the strainers, shower drain traps, and their holes. Clinical data were collected from nursing home medical records at baseline and each follow-up visit using the REDCap electronic data capture tools hosted by the National Healthcare Group [8]. 

Stool and environmental samples were sent to the laboratory within 24 h. Stool samples were inoculated onto CHROMID^®^ CARBA SMART agar and incubated overnight at 37^o^C. The environmental samples were incubated overnight in 5 ml tryptic soy broth with 10 µg meropenem and subsequently subcultured on CHROMID^®^ CARBA SMART agar. Positive cultures underwent matrix-assisted laser desorption ionization – time of flight (MALDI-TOF) for bacterial identification. Antibiotic susceptibility testing was performed on the Enterobacterales identified, adhering to Clinical and Laboratory Standards Institute (CLSI) guidelines. Check-Direct CPE^®^ (Check-Points) typing was carried out to detect carbapenemase genes (*bla*KPC, *bla*OXA-48, *bla*NDM, and *bla*VIM).

### Whole genome sequencing and analysis for determination of genomic linkage

Genomic DNA was extracted for each isolate and sequenced using Illumina technology [9]. Trimmed reads were de novo assembled using SPAdes Genome Assembler [10]. Bacterial core genome analysis was based on a previously published pipeline [11]. Bacterial linkage was established between two isolates if they shared the same ST, same CP-gene allele and had recombination-filtered pairwise SNP count below the BEAST-derived mutation rate threshold, assuming a Poisson distribution for the accumulation of mutation [11, 12]. A bacterial linkage cluster was defined as isolates that met bacterial linkage criteria with at least one other isolate in the cluster.

Plasmid identification was performed for all isolates using PlasmidSeeker and BLAST, against carbapenemase-gene allele-specific plasmid reference databases. Only candidate plasmid sequences that shared ≥ 90% k-mers with the isolate by PlasmidSeeker and had CP-gene-containing assembled contig present with coverage of ≥ 90% by BLAST were considered [11, 13]. Subsequently, plasmid linkage was established between two isolates if they shared at least one plasmid. Seventeen blaNDM-positive isolates were sequenced using long-read sequencing and hybrid-assembled using Unicycler 0.48 to further aid in determination of plasmid linkage [14]. A plasmid linkage cluster was defined as isolates that met plasmid linkage criteria with at least one other isolate in the cluster.

Details regarding genome sequencing, core genome analysis, determination of SNP thresholds, generation of plasmid sequence reference database and criteria for establishing genomic linkage are provided in the Supplementary Methods.

### Statistical methods

We compared clinical characteristics of known CPE carriers and residents with no history of CPE carriage using Fisher’s exact test for categorical variables and the Wilcoxon rank-sum test for continuous variables. The time at risk for CPE acquisition was defined as the period between the baseline stool sample collection date and week 12. CPE acquisition was defined as having a positive CPE stool sample after an initial negative screening at recruitment. CPE acquisition rate per 1000 patient-days and 95% confidence interval were calculated using the Mantel-Haenszel method. Data analysis was performed using Stata version 15.0.

### Ethics approval

The study was approved by the National Healthcare Group Domain Specific Review Board (2018/00439) before initiation.

## Result

**Fig. 1:**
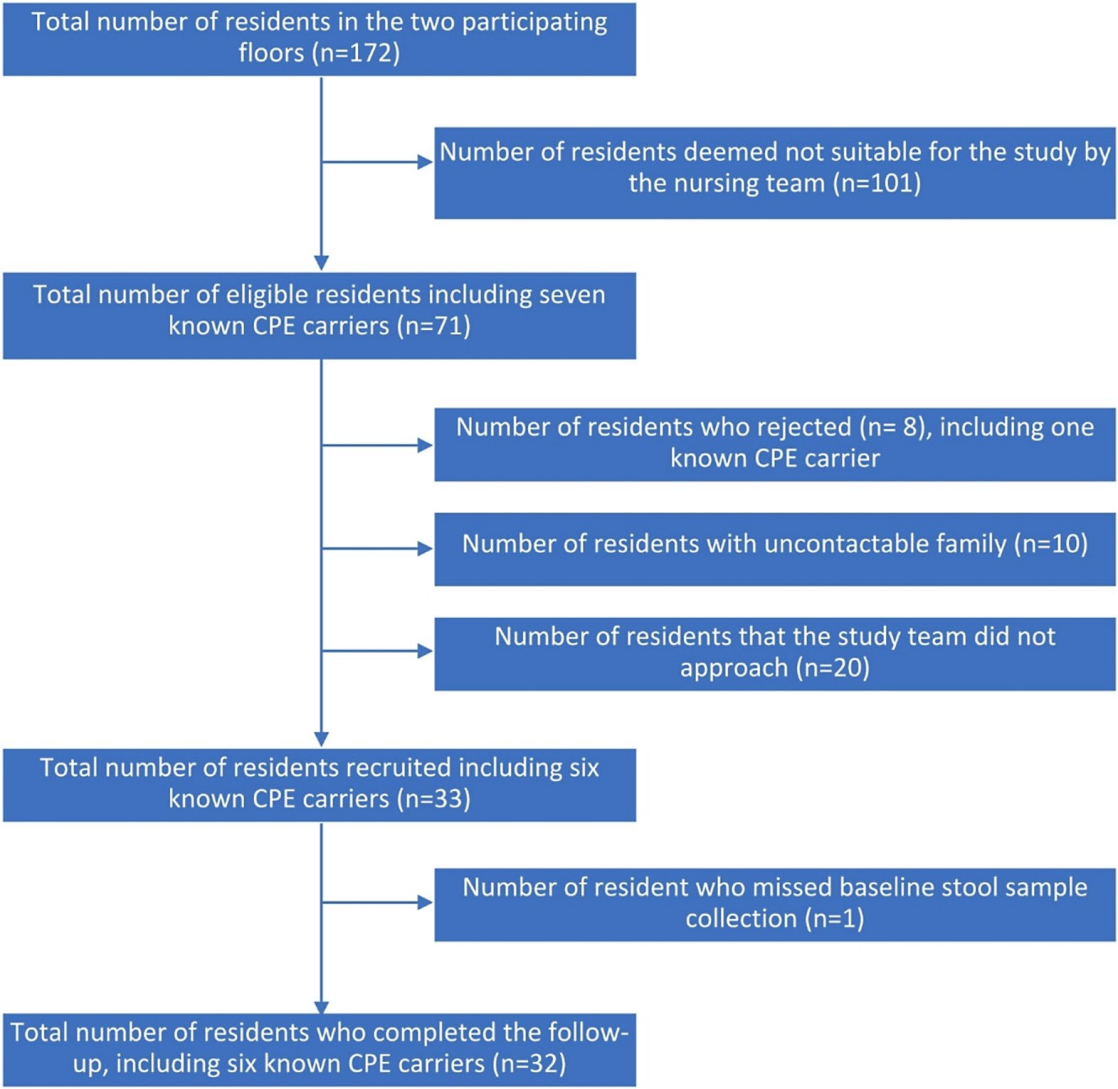
Recruitment and follow-up of subjects

Of the 172 residents, which included seven known CPE carriers, 32 residents including six known CPE carriers (five with NDM and one with OXA-48) were recruited and completed study follow up (Fig. 1). The six known CPE carriers tested positive for CPE during their acute hospital stay before admission to the nursing home (Supplementary Table S2). Among the study participants, 27 (84.4%) were female, and the median age was 77 (IQR: 69,90) (Table 1). Of the six known CPE carriers, five tested negative for CPE throughout the study, while one was colonized with OXA-48-producing *Escherichia coli*, *Citrobacter koseri*, *Klebsiella pneumoniae* at baseline, OXA-48-producing *Escherichia coli* and *Klebsiella pneumoniae* at week 2 and week 8, and OXA-48-producing *Escherichia coli*,* Citrobacter koseri* and *Klebsiella pneumoniae* at week 12. The location of the resident beds along with the layout of the nursing home, are provided in Supplementary Figure S1 and Supplementary Figure S2.

**Table 1:** Clinical characteristics of the residents who completed the follow-up

Baseline characteristics	Residents who were known CPE carriers *n* = 6 (%)	Residents who were not known to carry CPE *n* = 26 (%)	Total *n* = 32 (%)	Fisher’s exact test
Age, Median (IQR)	71 (56–78)	78 (72–92)	77 (69–90)	0.11^a^
Gender, Female	6 (100.00)	21 (80.77)	27 (84.38)	0.56
**Comorbidities**				
Chronic obstructive pulmonary disease	0 (0.00)	3 (11.54)	3 (9.38)	1.00
Cardiovascular disease	1 (16.67)	6 (23.08)	7 (21.88)	1.00
Chronic neurological disease	5 (83.33)	19 (73.08)	24 (75.00)	1.00
Malignant disease	1 (16.67)	3 (11.54)	4 (12.50)	1.00
Chronic kidney disease	0 (0.00)	5 (19.23)	5 (15.63)	0.56
Diabetes Mellitus	1 (16.67)	5 (19.23)	6 (18.75)	1.00
Chronic liver disease	0 (0.00)	1 (3.85)	1 (3.13)	1.00
**Ambulatory status**				
Ambulant with or without assistance	0 (0.00)	3 (11.54)	3 (9.38)	0.30
Ambulant with wheel-chair	4 (66.67)	20 (76.92)	24 (75.00)	
Bedbound	2 (33.33)	3 (11.54)	5 (15.63)	
**Invasive procedures at baseline**				
Nasogastric tube	3 (50.00)	2 (7.69)	5 (15.63)	0.03
Indwelling urinary catheter	1 (16.67)	1 (3.85)	2 (6.25)	0.35
Central line	0 (0.00)	0 (0.00)	0 (0.00)	Not applicable
**Medications at baseline**				
Antibiotics in the past 30 days	3 (50.00)	2 (7.69)	5 (15.63)	0.03
Proton pump inhibitors in the past 30 days	3 (50.00)	9 (34.62)	12 (37.50)	0.65

CPE, Carbapenemase-producing *Enterobacterales*; IQR, Interquartile range

^a^Wilcoxon rank-sum test

We collected a total of 164 environmental samples from sink strainers and shower drain traps. Of the 28 sink strainers, six (21.43%) were positive for CPE: five were *bla*NDM *Klebsiella pneumoniae*, and one was *bla*KPC *Klebsiella pneumoniae*. Different plasmids were detected in the same sink at each visit, although many of them were of the same plasmid cluster. All shower drain traps were negative for CPE throughout the study. The genotype and species of isolates obtained from residents and environment at baseline and follow-up visits are described in Supplementary Table S2.

During the 2699 patient-days follow-up, one resident (3.1%) acquired *Enterobacter cloacae* harboring a *bla*NDM-positive plasmid at week 12, resulting in an acquisition rate of 0.37 per 1000 person-days (95%CI: 0.05–2.63). The affected resident was wheelchair ambulant and was not hospitalized or exposed to antibiotics. The resident is known to have chronic pulmonary disease, dementia, and diabetes mellitus with chronic complications. The resident shared a room with 2 known CPE carriers, one of whom remained negative throughout the study, while the other tested positive for *Escherichia coli*,* Citrobacter koseri*,* Klebsiella pneumoniae* harboring *bla*_OXA−48_ gene. None of the study participants tested positive for *bla*NDM throughout the study. Environmental samples from the resident’s room and adjacent rooms were negative for CPE throughout the study. WGS analysis of available CPE isolates showed no bacterial or plasmid linkage between residents or between residents and the environment (Supplementary Table S3).

## Discussion

Our findings suggest that the use of standard precautions and unrestricted movement of CPE carriers may be sufficient to prevent CPE transmissions in nursing homes. However, one observed CPE transmission with no attributable source is concerning and warrants further investigation in a larger study. It is worth noting that one-fifth of the environment samples tested positive for CPE. Although no direct transmission was observed due to the lack of bacterial or plasmid linkage between residents and environment, the predominance of *bla*_NDM_* Klebsiella pneumoniae* in CPE-positive environmental samples (with only one exception), combined with the fact that five of the six known CPE carriers were known to carry NDM, suggests a potential indirect role of the environment in CPE transmission.

Compared to previous studies in Europe, where CPE prevalence in nursing homes was found to be low at less than 0.1%, [15, 16] the prevalence of known CPE carriers in the study site was 4.1% (7 residents out of a total of 172 residents) (Fig. 1). However, only one of the seven known CPE carriers remained positive during this study. Together with newly diagnosed CPE acquisition, the new prevalence was 1.2%.

Our study has several limitations. Firstly, the small sample size limits the statistical power and generalizability of our findings. The low number of participants, particularly the presence of only two patients with positive stool cultures during the study period, may have affected the robustness of our analyses. Additionally, only stool samples were collected to assess CPE colonization in the residents. This limitation may have affected our ability to detect colonization at other body sites, such as the skin, respiratory system, or wounds, which may have been more relevant for environmental contamination. Secondly, we only sampled sink strainers and shower drains potentially missing other environmental reservoirs. This limited environmental sampling may have contributed to the lack of direct evidence for CPE transmission from the environment within the facility. Lastly, the inability to assess the overall prevalence of the antibiotic utilization is another limitation of this pilot study. Although the one patient who acquired CPE was not on any antimicrobials, the overall antimicrobial utilization burden in the nursing home may have influenced the CPE acquisition. Given these limitations, including the short three-month follow-up period, our findings should be interpreted with caution.

This pilot study found a low CPE acquisition rate among nursing home residents and no CPE transmission between residents, despite one-fifth of environmental samples testing positive for CPE. These findings suggest that standard precautions and unrestricted movement of CPE carriers may be sufficient to control CPE transmission in the nursing home setting. Larger studies with more extensive environmental sampling and longer follow-up periods are needed to confirm the effectiveness of these measures in controlling CPE transmission in nursing homes.

## Electronic supplementary material

Below is the link to the electronic supplementary material.

Supplementary Material 1

## Data Availability

The data that support the findings of this study are available from the nursing home electronic medical record database but restrictions apply to the availability of these data, which were used under license for the current study, and so are not publicly available. Data are however available from the authors upon reasonable request and with permission of the nursing home and Domain Specific Review Board.

## Abbreviations

CPE: Carbapenemase-producing Enterobacterales
IPC: Infection Prevention & Control
IQR: Interquartile range
NIPC: National Infection Prevention & Control
WGS: Whole genome sequencing
