# Supplementary material for: Impact of standard precautions and unrestricted movements of carbapenemase-producing Enterobacterales (CPE) carriers on CPE transmission in a nursing home in Singapore: a prospective cohort study
Source: Antimicrob Resist Infect Control. 2025 Apr 28;14:38. doi: 10.1186/s13756-025-01554-1 (PMC12039280; doi:10.1186/s13756-025-01554-1)
Supplement: Supplementary file 1 — Supplementary Material 1 [file 13756_2025_1554_MOESM1_ESM.docx]

**Supplementary Method**

***Whole-genome sequencing and assignment of genomic species and carbapenemase gene***

Genomic DNA for each isolate was extracted using MagNA Pure Compact (Roche Applied Science, Germany), and sequenced on the Illumina HiSeq platform to generate 2x150bp paired-end reads. The raw reads were adapter trimmed, quality trimmed and filtered using BBDuk (version 38.11; with parameters: ktrim=r k=23 mink=11 hdist=1 qtrim=rl trimq=20 minavgquality=20; https://archive.jgi.doe.gov/data-and-tools/software-tools/bbtools/). *De novo* assembly of the trimmed reads was performed using SPAdes Genome Assembler (with the "--careful" option).^1^ For each assembly, only contigs with length at least 1000 bps were considered for subsequent analysis. Two assemblies were not further analysed due to abnormal assembly sizes, resulting in 35 isolates successfully assembled for downstream analysis.

Bacterial species were assigned for each isolate using Kraken (version 1.0, with default parameters) on the trimmed reads, and sequence-type (ST) was determined using MLST (version 2.16.2) on the assemblies.^2,3^ Genomic carbapenemase gene (CP-gene) assignments were performed using CGE Resfinder.^4^ Six isolates had no carbapenemase gene detected by both lab- and genomic-based methods, and hence were removed, resulting in 29 isolates included for downstream analysis (Supplementary Table S3).

***Bacterial core genome analysis***

The isolates were grouped according to their ST assignments. As there were only 2 isolates for *K. pneumoniae* ST903, they were combined with isolates of *K. pneumoniae* ST499 to form a single ST group for the purpose of core genome analysis. Gene annotation was performed using Prokka v1.12,^5^ and the annotated assemblies of all isolates for a given ST group were used as input for Roary (v1.007002), with BLASTP identity threshold set to 95% and generating a core gene alignment of genes that span 95% of the isolates for the ST.^6^

For each ST group, the single-nucleotide polymorphisms (SNPs) based on the core gene alignment were called using SNP-sites (v2.4.1), with the consensus sequence of the alignment as a reference.^7^ To validate the alignment-based SNP calls, mapping of the short-read data followed by SNP calling was also performed using Snippy (v3.1; https://github.com/tseemann/snippy). Only SNPs in the core gene alignment that were found to be supported by the mapping-based SNP calling were retained in the alignment, while unvalidated SNPs were masked.

The resulting core gene alignment for each ST group was "padded" with invariant sites in a proportion according to the GC content and length of the selected reference genome (NCBI GenBank accession numbers: NC_012731.1 for *K. pneumoniae* (strain NTUH-K2044),^8^ NC_013654.1 for *E. coli*,^9^ NC_009792.1 for *C. koseri*.^10^ The "padded" alignments were then filtered to remove recombinant regions using Gubbins (v2.3.4).^11^

***Determination of single-nucleotide polymorphism (SNP) threshold for bacterial linkage***

The mutation rates for each species were obtained from another study,^12^ in which the mutation rate for *K. pneumoniae* and *E. coli* were estimated based on datasets consisting of *K. pneumoniae* ST14 (60 isolates) and *E. coli* ST131 (65 isolates) respectively, and the mutation rate for *C. koseri* was estimated based on a dataset of *C. freundii* ST 22 (28 isolates) due to the limited number of *C. koseri* isolates. Three independent BEAST runs were performed for each dataset, with the following parameters adapted from a previously published study: site model set as bModelTest, strict clock rate, prior set as "coalescent constant population", and Monte Carlo Markov chain (MCMC) iterations set as 300 million.^9,13^ The combined effective sample sizes (ESS) for all parameters was greater than 200. The resulting mutation rate thresholds applied for determining bacterial linkage (substitutions/genome/year) were 1.009 for *K. pneumoniae*, 0.33 for *E.coli* and 2.72 for *C.koseri*.

Bacterial linkage was established between two isolates if they shared the same ST, same CP-gene allele and had pairwise SNP count (based on the recombination-filtered core gene alignments) below the BEAST-derived mutation rate threshold, assuming a Poisson distribution for the accumulation of mutations.^14^ A bacterial linkage cluster was defined as isolates that met bacterial linkage criteria with at least one other isolate in the cluster.

***Long-read sequencing and determination of plasmid linkage***

Fully-circularized carbapenemase-encoding plasmid genome sequences, which are plasmid sequences that have been assembled as a single, continuous, circular contig, and determined to harbour a carbapenemase gene, were made available from an ongoing sequencing project using Oxford Nanopore Technologies (ONT) long-read sequencing. The libraries for long-read sequencing were prepared using the ONT Rapid Barcoding Kit (RBK004) and sequenced on R9.4.1 flowcells on the GridION platform. Hybrid assembly of the ONT long-read sequence data with Illumina short-read data was performed using the Unicycler 0.48 hybrid assembler with default parameters.^15^

Reference databases of plasmid genome sequences for each CP-gene allele were generated from (i) complete plasmid genome sequences downloaded from NCBI RefSeq and (ii) fully-circularized carbapenemase-encoding plasmid genome sequences (as defined above) obtained from the long-read sequencing assemblies as described above. The databases were then filtered to retain only plasmid genome sequences that contained the CP-gene allele with 100% identity and 100% gene coverage.

Plasmid identification was performed for all isolates using PlasmidSeeker (v0.1; 2017-04-21) with default parameters, against the CP-gene allele-specific reference databases.^16^ For each isolate, the CP-gene-containing contig from the assembly was BLAST-aligned (as query) against the candidate plasmids obtained by PlasmidSeeker. Only candidate plasmids that shared ≥90% k-mers with the isolate (kcov) and had CP-gene-containing contig present with coverage (qcov) of ≥90% were considered. Subsequently, plasmid linkage was established between two isolates if they shared at least one plasmid.

Seventeen *bla*NDM-positive isolates were sequenced using long-read sequencing and hybrid-assembled as described above to further aid in determination of plasmid linkage. The fully-circularized carbapenemase-encoding plasmid sequence were identified, and plasmid linkage was determined by direct visualization guided by pairwise BLASTN showing pairwise identity and alignment coverage more than 99%.

A plasmid linkage cluster was defined as isolates that met plasmid linkage criteria with at least one other isolate in the cluster.

**Table S1. Follow-up schedule and data collection for the recruited residents**

| Time since recruitment | Baseline | Week 2 | Week 8 | Week 12 |
| --- | --- | --- | --- | --- |
| Demographic details | ⚫ |  |  |  |
| Comorbidities | ⚫ |  |  |  |
| Ambulatory status | ⚫ | ⚫ | ⚫ | ⚫ |
| Invasive procedures | ⚫ | ⚫ | ⚫ | ⚫ |
| Medication history | ⚫ | ⚫ | ⚫ | ⚫ |
| Stool and environmental sample collection | ⚫ | ⚫ | ⚫ | ⚫ |

| **Sample source** | **CPE status before recruitment (Date of sample collection)** | **Baseline** | | **Week 2** | | **Week 8** | | **Week 12** | |
| --- | --- | --- | --- | --- | --- | --- | --- | --- | --- |
|  |  | **Genotype** | **Species** | **Genotype** | **Species** | **Genotype** | **Species** | **Genotype** | **Species** |
| Resident 1 | NDM (02/10/18) | Negative | Negative | Negative | Negative | Negative | Negative | Negative | Negative |
| Resident 2 | NDM  (27/09/18) | Negative | Negative | Negative | Negative | Negative | Negative | Negative | Negative |
| Resident 3 | NDM  (18/07/18) | Negative | Negative | Negative | Negative | Negative | Negative | Negative | Negative |
| Resident 4 | NDM  (20/05/15) | Negative | Negative | Negative | Negative | Negative | Negative | Negative | Negative |
| Resident 5 | NDM  (19/07/16) | Negative | Negative | Negative | Negative | Negative | Negative | Negative | Negative |
| Resident 6 | OXA-48  (17/01/17) | OXA-48 | *E. coli, K. pneumoniae*, C. koseri* | OXA-48 | *E. coli, K. pneumoniae* | OXA-48 | *E. coli, K. pneumoniae* | OXA-48 | *E. coli, K. pneumoniae, C. koseri* |
| Resident 7 | Negative | Negative | Negative | Negative | Negative | Negative | Negative | NDM | *Enterobacter cloacae* |
| Sink steel trap 1 | Not known | NDM | *K. pneumoniae* | NDM | *K. pneumoniae* | NDM | *K. pneumoniae* | NDM | *K. pneumoniae* |
| Sink steel trap 2 | Not known | NDM | *K. pneumoniae* | NDM | *K. pneumoniae* | NDM | *K. pneumoniae* | NDM | *K. pneumoniae* |
| Sink steel trap 3 | Not known | Negative | Negative | Negative | Negative | Negative | Negative | NDM | *K. pneumoniae* |
| Sink steel trap 4 | Not known | Negative | Negative | KPC | *K. pneumoniae* | Negative | Negative | Negative | Negative |
| Sink steel trap 5 | Not known | NDM | *K. pneumoniae* | NDM | *K. pneumoniae* | NDM | *K. pneumoniae* | NDM | *K. pneumoniae* |
| Sink steel trap 6 | Not known | NDM | *K. pneumoniae** | NDM | *K. pneumoniae* | NDM | *K. pneumoniae* | NDM | *K. pneumoniae* |

**Table S2. Genotype and species grown from the samples from residents and environment at baseline and follow-up visits**

*The isolate was excluded from the bacterial and plasmid clustering analysis due to problems with genome assembly

**Table S3. Sequence type, bacterial linkage cluster, and plasmid linkage cluster of the bacterial isolates from the collected samples**

| **Sample ID** | **Sample type** | **Species** | **Sequence type (ST)** | **Carbapenemase gene** | **Bacterial linkage cluster** | **Plasmid linkage cluster** |
| --- | --- | --- | --- | --- | --- | --- |
| 1 | Sink 5 Baseline | *Klebisella pneumoniae* | 252 | NDM-1 | Cluster_B1 | Cluster_P1 |
| 2 | Sink 5 Week 2 | *Klebisella pneumoniae* | 252 | NDM-1 | Cluster_B1 | Cluster_P1 |
| 3 | Resident 6 Week 2 | *Escherichia coli* | 162 | OXA-48 | Cluster_B2 | Cluster_P2 |
| 4 | Sink 6 Week 2 | *Klebisella pneumoniae* | 252 | NDM-1 | Cluster_B1 | Cluster_P1 |
| 5 | Sink 5 Week 12 | *Klebisella pneumoniae* | 252 | NDM-1 | Cluster_B1 | Cluster_P1 |
| 6 | Sink 6 Week 12 | *Klebisella pneumoniae* | 252 | NDM-1 | Cluster_B1 | Cluster_P1 |
| 7 | Resident 6 Week 2 | *Klebisella pneumoniae* | 499 | OXA-48 | Cluster_B3 | Cluster_P2 |
| 8 | Resident 6 Week 8 | *Klebisella pneumoniae* | 499 | OXA-48 | Cluster_B3 | Cluster_P2 |
| 9 | Resident 6 Week 12 | *Klebisella pneumoniae* | 499 | OXA-48 | Cluster_B3 | Cluster_P2 |
| 10 | Sink 1 Baseline | *Klebisella pneumoniae* | 2133 | NDM-1 | Cluster_B4 | NA |
| 11 | Sink 1 Week 2 | *Klebisella pneumoniae* | 2133 | NDM-1 | Cluster_B4 | Cluster_P1 |
| 12 | Sink 2 Week 8 | *Klebisella pneumoniae* | 2133 | NDM-1 | Cluster_B5 | Cluster_P1 |
| 13 | Sink 2 Week 12 | *Klebisella pneumoniae* | 2133 | NDM-1 | Cluster_B5 | Cluster_P1 |
| 14 | Sink 1 Week 8 | *Klebisella pneumoniae* | 903 | NDM-1 | Cluster_B6 | NA |
| 15 | Resident 6 Week 12 | *Escherichia coli* | 162 | OXA-48 | Cluster_B2 | Cluster_P2 |
| 16 | Sink 1 Week 12 | *Klebisella pneumoniae* | 903 | NDM-1 | Cluster_B6 | NA |
| 17 | Sink 2 Baseline | *Klebisella pneumoniae* | 2133 | NDM-1 | Cluster_B7 | Cluster_P1 |
| 18 | Sink 2 Week 2 | *Klebisella pneumoniae* | 2133 | NDM-1 | Cluster_B7 | NA |
| 19 | Resident 6 Baseline | *Escherichia coli* | 162 | OXA-48 | NA | Cluster_P2 |
| 20 | Sink 4 Week 2 | *Klebisella pneumoniae* | 45 | KPC-2 | NA | NA |
| 21 | Resident 6 Week 8 | *Escherichia coli* | 162 | OXA-48 | NA | Cluster_P2 |
| 22 | Resident 6 Week 12 | *Escherichia coli* | 162 | OXA-48 | NA | Cluster_P2 |
| 23 | Resident 6 Week 12 | *Citrobacter koseri* | - | OXA-48 | NA | Cluster_P2 |
| 24 | Resident 7 Week 12 | *Enterobacter cloacae* | 171 | NDM-1 | NA | NA |
| 25 | Sink 5 Week 8 | *Klebisella pneumoniae* | 252 | NDM-1 | NA | Cluster_P1 |
| 26 | Sink 6 Week 8 | *Klebisella pneumoniae* | 1040 | NDM-1 | NA | Cluster_P1 |
| 27 | Sink 3 Week 12 | *Klebisella pneumoniae* | - | NDM-1 | NA | NA |
| 28 | Resident 6 Baseline | *Citrobacter koseri* | - | OXA-48 | NA | Cluster_P2 |
| 29 | Resident 6 Baseline | *Citrobacter koseri* | - | OXA-48 | NA | Cluster_P2 |

NA, Not available which means the sample was not part of any bacterial cluster or plasmid cluster; Resident 6, Known CPE carrier; Resident 7, Resident who acquired NDM producing Enterobacter cloacae at week 12

**Figure S1. Layout of the floor that housed female residents**


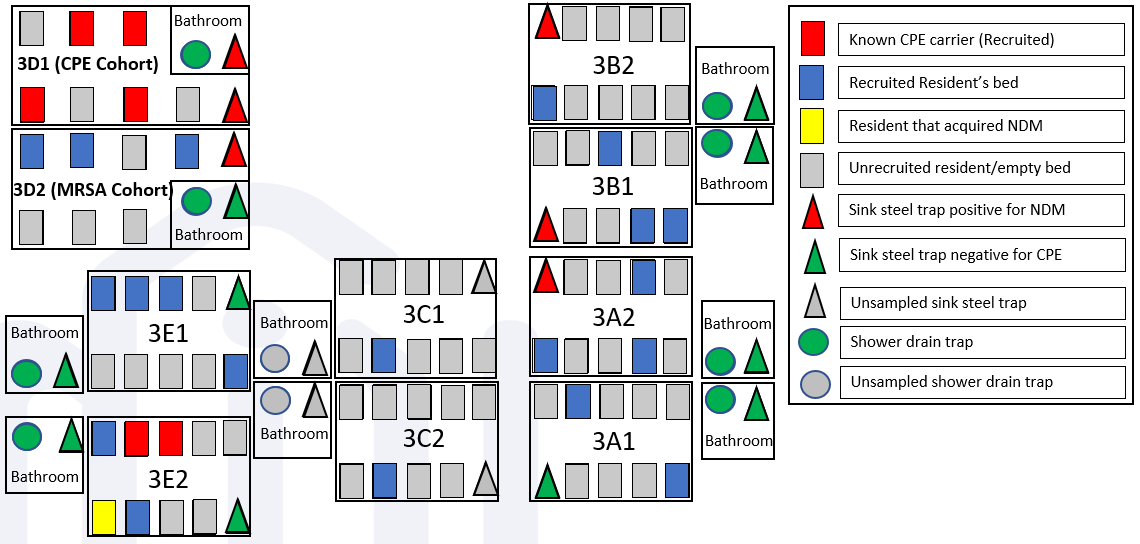


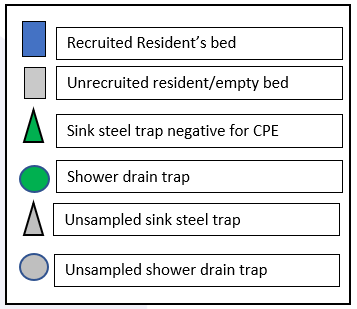
**Figure S2. Layout of the floor that housed male residents**
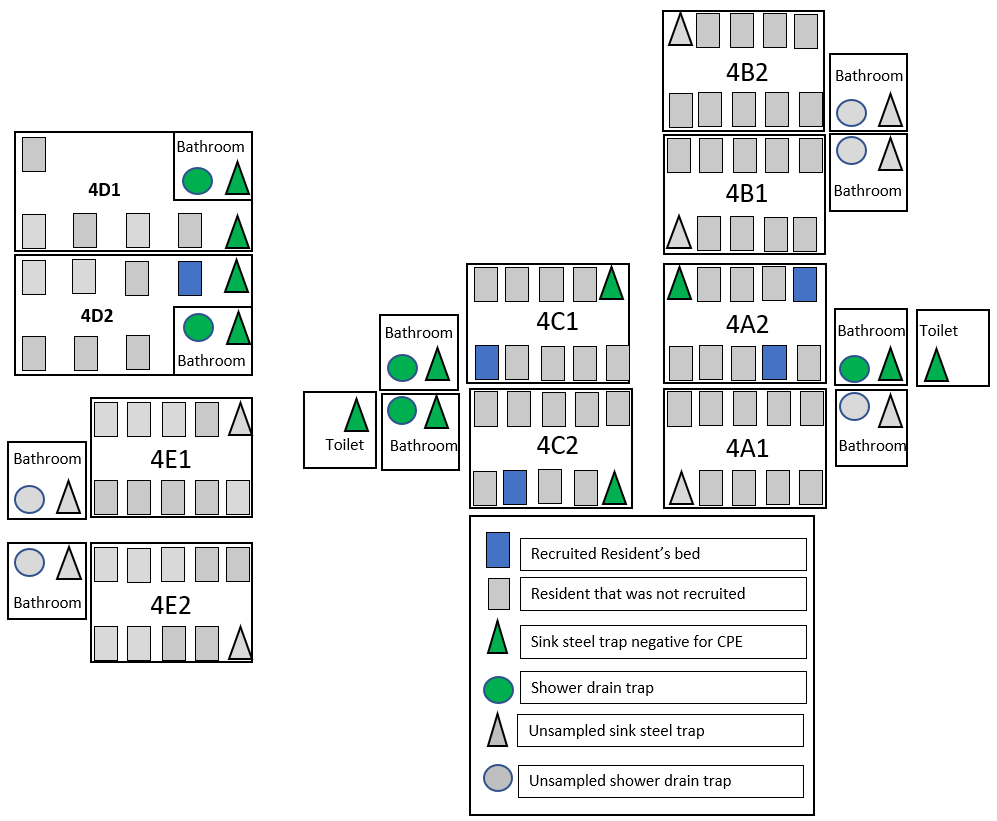


**Supplementary references**

**1.** Bankevich A, Nurk S, Antipov D, et al. SPAdes: a new genome assembly algorithm and its applications to single-cell sequencing. *J Comput Biol* 2012;19:455-477.

**2.** Wood DE, Salzberg SL. Kraken: ultrafast metagenomic sequence classification using exact alignments. *Genome Biol* 2014;15:R46.

**3.** Urwin R, Maiden MC. Multi-locus sequence typing: a tool for global epidemiology. *Trends Microbiol* 2003;11:479-487.

**4.** Bortolaia V, Kaas RS, Ruppe E, et al. ResFinder 4.0 for predictions of phenotypes from genotypes. *J Antimicrob Chemother* 2020;75:3491-3500.

**5.** Seemann T. Prokka: rapid prokaryotic genome annotation. *Bioinformatics* 2014;30:2068-2069.

**6.** Page AJ, Cummins CA, Hunt M, et al. Roary: rapid large-scale prokaryote pan genome analysis. *Bioinformatics* 2015;31:3691-3693.

**7.** Page AJ, Taylor B, Delaney AJ, et al. SNP-sites: rapid efficient extraction of SNPs from multi-FASTA alignments. *Microb Genom* 2016;2:e000056.

**8.** Lam MMC, Wyres KL, Duchene S, et al. Population genomics of hypervirulent Klebsiella pneumoniae clonal-group 23 reveals early emergence and rapid global dissemination. *Nat Commun* 2018;9:2703.

**9.** Stoesser N, Sheppard AE, Pankhurst L, et al. Evolutionary History of the Global Emergence of the Escherichia coli Epidemic Clone ST131. *mBio* 2016;7:e02162.

**10.** Ekwanzala MD, Dewar JB, Kamika I, Momba MNB. Genome sequence of carbapenem-resistant Citrobacter koseri carrying bla(OXA-181) isolated from sewage sludge. *J Glob Antimicrob Resist* 2020;20:94-97.

**11.** Croucher NJ, Page AJ, Connor TR, et al. Rapid phylogenetic analysis of large samples of recombinant bacterial whole genome sequences using Gubbins. *Nucleic Acids Res* 2015;43:e15.

**12.** Marimuthu K, Venkatachalam I, Koh V, et al. Whole genome sequencing reveals hidden transmission of carbapenemase-producing Enterobacterales. *Nat Commun* 2022;13:3052.

**13.** Bouckaert RR, Drummond AJ. bModelTest: Bayesian phylogenetic site model averaging and model comparison. *BMC Evol Biol* 2017;17:42.

**14.** Eyre DW, Walker AS, Freeman J, et al. Short-term genome stability of serial Clostridium difficile ribotype 027 isolates in an experimental gut model and recurrent human disease. *PLoS One* 2013;8:e63540.

**15.** Wick RR, Judd LM, Gorrie CL, Holt KE. Unicycler: Resolving bacterial genome assemblies from short and long sequencing reads. *PLoS Comput Biol* 2017;13:e1005595.

**16.** Roosaare M, Puustusmaa M, Mols M, Vaher M, Remm M. PlasmidSeeker: identification of known plasmids from bacterial whole genome sequencing reads. *PeerJ* 2018;6:e4588.
